# Obstetrics

**Published:** 1861-12

**Authors:** 


**OBSTETRICS.**—The Messrs. Wood, the great medical publishers of the United States, have, with commendable and courageous enterprise, issued a beautiful octavo volume of 761 pages, on "The Principles and Practice of Obstetrics, by Gunning S. Bedford, A.M., M.D., author of 'Clinical Lectures on the Diseases of Women and Children,'" and the distinguished Professor of Obstetrics, etc., in the University of New-York. It is printed in handsome style, and illustrated by four colored lithograph plates, and ninety-nine engravings. It is a complete and able exposition of the science and practice of midwifery, and is destined to be a text-book and a standard volume in American medical literature. But Professor Bedford's name alone would sell the book, so that the Messrs. Wood, after all, were not quite so brave as at first sight might have seemed, in bringing out so costly a work in times like these, when so many publishers would be but too happy to sell what they have on hand. They keep in their large establishment the fullest and most complete assortment of medical works of real value in America, both English and French, and at very low prices.

